# μ-Opioid Receptor Antibody Reveals Tissue-Dependent Specific Staining and Increased Neuronal μ-Receptor Immunoreactivity at the Injured Nerve Trunk in Mice

**DOI:** 10.1371/journal.pone.0079099

**Published:** 2013-11-22

**Authors:** Yvonne Schmidt, Claire Gavériaux-Ruff, Halina Machelska

**Affiliations:** 1 Klinik für Anästhesiologie und operative Intensivmedizin, Freie Universität Berlin, Charité- Universitätsmedizin Berlin, Campus Benjamin Franklin, Berlin, Germany; 2 Institut de Génétique et de Biologie Moléculaire et Cellulaire, UdS Université de Strasbourg, Strasbourg, Inserm, U964; CNRS, UMR7104, Illkirch, France; University of Kentucky Medical Center, United States of America

## Abstract

Neuropathic pain is a debilitating chronic disease often resulting from damage to peripheral nerves. Activation of opioid receptors on peripheral sensory neurons can attenuate pain without central nervous system side effects. Here we aimed to analyze the distribution of neuronal μ-opioid receptors, the most relevant opioid receptors in the control of clinical pain, along the peripheral neuronal pathways in neuropathy. Hence, following a chronic constriction injury of the sciatic nerve in mice, we used immunohistochemistry to quantify the μ-receptor protein expression in the dorsal root ganglia (DRG), directly at the injured nerve trunk, and at its peripheral endings in the hind paw skin. We also thoroughly examined the μ-receptor antibody staining specificity. We found that the antibody specifically labeled μ-receptors in human embryonic kidney 293 cells as well as in neuronal processes of the sciatic nerve and hind paw skin dermis, but surprisingly not in the DRG, as judged by the use of μ/δ/κ-opioid receptor knockout mice. Therefore, a reliable quantitative analysis of μ-receptor expression in the DRG was not possible. However, we demonstrate that the μ-receptor immunoreactivity was strongly enhanced proximally to the injury at the nerve trunk, but was unaltered in paws, on days 2 and 14 following injury. Thus, μ-opioid receptors at the site of axonal damage might be a promising target for the control of painful neuropathies. Furthermore, our findings suggest a rigorous tissue-dependent characterization of antibodies' specificity, preferably using knockout animals.

## Introduction

Neuropathic pain can result from peripheral nerve injuries such as amputation, entrapment, or compression. Such neuropathies trigger maladaptive alterations in the nervous system leading to peripheral and central sensitization that underlie transition to chronic pain [1]. Therapy with classical opioids predominantly acting at μ-opioid receptors is limited by detrimental effects, including respiratory failure, nausea, dependence, and addiction mediated in the central nervous system [2]. Importantly, these side effects can be avoided by activating opioid receptors on peripheral sensory neurons. Peripheral analgesic effects of opioids in neuropathic conditions were tested in animal models utilizing ligations of the nerve trunk [3]. Yet, opioids were commonly applied to tissues remote from the nerve lesion site, i.e. to paws innervated by damaged nerves, leading to partial attenuation [4]–[8] or no improvement of hypersensitivity [9]–[12]. Interestingly, opioid peptides derived from immune cells accumulating at the site of nerve injury [13], [14] or exogenous μ-receptor agonists injected at this site [15] reversed mechanical or thermal hypersensitivity, suggesting that opioid receptors at the nerve injury site are functional.

However, the expression of peripheral μ-opioid receptors in neuropathy was mostly assessed in the dorsal root ganglia (DRG). Depending on the nerve damage type or the DRG level, the number of μ-receptor-immunoreactive cells examined with immunohiostochemistry was either unaltered [16], [17], increased [15], or decreased [11], [17]–[19]. In addition, the μ-receptor immunoreactivity assessed with Western blot was elevated [17], [20] or diminished [17]. Nevertheless, the opioid receptor level in the DRG might not be predictive for peripheral opioid analgesia in neuropathy. The μ-receptor immunoreactivity was enhanced at the nerve injury site [15], while it was either increased [20], [21] or decreased [17] in the hind paw skin innervated by the damaged nerve. Still, the receptor cellular sources in these tissues were so far not identified.

Our aim was to analyze the expression of μ-opioid receptor protein along the peripheral neuronal pathways, including DRG, nerve trunk and its peripheral terminals, which are the most relevant to peripheral opioid analgesia, in neuropathy. As a model of such condition, we used a chronic constriction injury (CCI) of the sciatic nerve in mice. Furthermore, we considered the current controversy about the specificity of opioid receptor antibodies [22], [23]. Accordingly, we used untransfected and μ-receptor transfected human embryonic kidney (HEK) 293 cells, as well as DRG, sciatic nerve, and paw tissue of wild type and opioid receptor knockout mice, and performed detailed control experiments to ensure a specific identification of μ-receptors.

## Methods

### Transfection of HEK 293 cells

HEK 293 cells (Leibniz Institute DSMZ, Germany) were transiently transfected with plasmids containing the full-length cDNA (approximately 2 µg) of the mouse μ-opioid receptor or the mouse δ-opioid receptor fused with enhanced green fluorescent protein (eGFP) [24]. Transfection was done with X-tremeGENE HP DNA transfection reagent, following the protocol of the manufacturer (Roche, Basel, Switzerland).

### Animals

Experiments were performed according to the guidelines of the International Association for the Study of Pain [25] and were approved by the State animal care committee (Landesamt für Gesundheit und Soziales, Berlin). They were carried out in male mice (25–30 g) that were either C57BL/6J wild type (Harlan Laboratories, Horst, Netherlands), or triple μ/δ/κ-opioid receptor knockout on a 129 (50%)/C57BL/6J (50%) genetic background, and the corresponding 129 (50%)/C57BL/6J (50%) wild type [26]. All mice were bred at the Charité, Berlin, and were kept in groups of 3–5 per cage, with free access to food and water, in environmentally controlled conditions (12 h light/dark schedule; 22±0.5°C; humidity 60–65%).

### Staining specificity of μ-opioid receptor antibody

After transfection with μ- or δ-opioid receptor cDNA (see above), HEK 293 cells were washed in phosphate buffered saline (PBS), fixed in 4% paraformaldehyde and 4% sucrose in PBS for 15 min at room temperature, again washed, and permeabilized in 0.25% TritonX-100 in PBS for 5 min. After washing, cells were blocked with 10% bovine serum albumin (BSA) in PBS for 30 min at 37°C, and incubated with a rabbit μ-receptor polyclonal primary antibody (1∶800; Ab10275, Abcam, Cambridge, UK) in 3% BSA/PBS for 2 h at 37°C. The antibody was designed to recognize the C terminal amino acids 384–398 (i.e. NHQLENLEAETAPLP) of the rat μ-receptor 1, but it also reacts with the identical sequence present in the C terminus of the mouse μ-receptor. Of all three opioid receptors (μ, δ, κ), this sequence is unique for the μ-receptor. After washing, the sections were incubated with a goat anti-rabbit texas red-conjugated secondary antibody (Vector Laboratories, Burlingame, CA) in 3% BSA/PBS for 45 min at 37°C, again washed, and mounted in Mowiol (Merck, Darmstadt, Germany).

μ/δ/κ-Opioid receptor knockout (n = 4) and the corresponding genetic background wild type mice (n = 4) were genotyped by PCR on genomic DNA samples obtained from tails, using specific primers (Table 1). All mice were deeply anesthetized with isoflurane (Abbott, Wiesbaden, Germany) and perfused transcardially with 0.1 M PBS (pH 7.4), followed by ice-cold 0.1 M PBS containing 4% paraformaldehyde (pH 7.4) (fixative solution). The lumbar 4 and 5 DRG, the sciatic nerve parts (8–10 mm-long), the skin with subcutaneous tissue from the plantar surface of hind paws, and the lumbar enlargement of the spinal cord were isolated and postfixed in the fixative solution for 2 h at 4°C, cryoprotected in 30% sucrose at 4°C overnight, embedded in OCT compound (Miles Inc. Elkhart, IN), and frozen at −80°C. The DRG were additionally incubated (approximately 30 s) in ice-cold 2-methyl-butane before embedding. Ten µm-thick sections were prepared from the DRG and longitudinally cut sciatic nerves, while 12 µm-thick sections were prepared from longitudinally cut paw tissue and transversely cut spinal cord, using a cryostat. The sections were mounted on gelatin-coated slides.

**Table 1:** Primer sequences used for PCR to genotype μ/δ/κ-opioid receptor knockout (KO) and the corresponding genetic background wild type (WT) mice.

Receptor	Product size	Primer	Sequence (5′- 3′)
μ	WT: 648 bp	Forward	GAGTTAGGAGAATCAGGAGTTCAAG
	KO: 422 bp	Reverse	TGCCATGAACATTACGGGCAGAC
		Forward middle	ACCGCTTCCTCGTGCTTTACGGTA
δ	WT: 1035 bp	Forward	GACCACGTGGTGCGCGCAGC
	KO: 591 bp	Reverse	AGAACACGCAGCACAAAGACTGG
		Forward middle	ACCGCTTCCTCGTGCTTTACGGTA
κ	WT: 290 bp	Forward	TTCTCGCTTTCCAGCTGCAGC
	KO: 580 bp	Reverse	CCTGAACTCACCGGATGATGACA
		Forward middle	ACCGCTTCCTCGTGCTTTACGGTA

To verify the expression of μ-receptors in neurons, the sections were stained with the μ-receptor antibody (1∶800; described above) and an antibody to pan-neuronal marker protein gene product 9.5 (PGP 9.5; 1∶800; Abcam). The sections were exposed to the blocking solution for 1 h. They were subsequently incubated overnight with the primary antibody solution. After washing, the sections were incubated for 1 h with texas red-conjugated goat anti-rabbit (1∶200; Vector Laboratories) or Alexa Flour® donkey anti-rabbit (1∶1000; Invitrogen, Carlsbad, USA), and fluorescein isothiocyanate (FITC)-conjugated goat anti-chicken (1∶200; GeneTex, Irvine, USA) or Alexa Flour® donkey anti-chicken (1∶1000; Jackson ImmunoResearch, Pennsylvania, USA) secondary antibodies, washed in PBS, and mounted in Mowiol, as previously [13].

Additional DRG sections were incubated for 45 min in PBS with 0.5% H_2_O_2_ and 45% methanol to block endogenous peroxidase. To prevent nonspecific binding, the sections were incubated for 60 min in PBS containing 0.3% Triton X-100, 1% BSA, and 5% goat serum (blocking solution). The sections were then incubated overnight with the μ-receptor antibody (1∶1500), washed, and stained with a vectastain avidin-biotin peroxidase complex using goat anti-rabbit biotinylated secondary antibody (Vector Laboratories). After washing, the sections were stained with 3′, 3′-diaminobenzidine (DAB) tetrahydrochloride (Vector Laboratories) for 30–60 s, washed in tap water, dehydrated in alcohol, cleared in xylene, and mounted in Entellan (Merck). Additional staining was performed after omission of the primary antibody.

### Nerve injury

CCI was induced in deeply isoflurane-anesthetized C57BL/6J mice by exposing the sciatic nerve at the level of the right mid-thigh and placing three loose silk ligatures (4/0) around the nerve. The wound was closed with silk sutures. Sham operation was performed in a similar manner, but without nerve ligation [13]. The following experiments were performed on days 2 and 14 after surgeries, which respectively represent early and later stages of CCI-induced neuropathic pain, examined in our previous studies [13], [14].

### Immunostaining of μ-opioid receptors following nerve injury

On days 2 and 14 after CCI or sham operation, animals were deeply anesthetized and perfused transcardially, as described above. The lumbar 4 and 5 DRG from ipsi- and contralateral sides to the CCI, parts (8–10 mm-long) of the injured and contralateral sciatic nerves, and skin with subcutaneous tissue from both hind paws were isolated. Parts of injured nerves included the ligation site and sites proximal and distal to it. Corresponding tissues were also obtained from sham-operated and naïve mice. All tissues were postfixed, cryoprotected, embedded in OCT compound, and frozen at -80°C, as described above (see “Staining specificity of μ-opioid receptor antibody”).

μ-Receptor staining using DAB in the DRG as well as using immunofluorescence in the nerve trunk and the hind paw skin was performed as described above (see “Staining specificity of μ-opioid receptor antibody”), except that PGP 9.5 was not stained. The sections following these staining procedures were used for quantitative analysis of μ-receptor expression described below. Additional staining was performed following omission of the μ-receptor primary antibody or preabsorption of the primary antibody with the μ-receptor immunizing peptide (5–10-fold excess, preincubation for 3 h).

Additionally, to verify the neuronal expression of μ-receptors, the sections of DRG ipsilateral to the CCI (on days 2 and 14 after injury; n = 3 mice per time point) were incubated overnight with the μ-receptor antibody (1∶800) alone and in combination with isolectin B4 (IB4) FITC-conjugated (1∶150; Sigma-Aldrich, St. Louis, USA), chicken neurofilament 200 (NF200; 1∶500; Chemicon, Billerica, USA), or guinea pig α-calcitonin gene-related peptide (CGRP; 1∶800; Bachem, Bubendorf, Switzerland) antibodies. After washing, the sections were incubated with texas red-conjugated goat anti-rabbit and goat anti-guinea pig, or FITC-conjugated goat anti-chicken secondary antibodies (1∶200; Vector Laboratories and GeneTex). Thereafter, the sections were washed in PBS, mounted in Mowiol, and viewed under a fluorescence microscope (Zeiss) with appropriate filters.

### Quantification of μ-opioid receptor immunostaining following nerve injury

Tissues from 5–6 mice were used per each experimental condition, i.e. naïve, sham operation, and CCI (as described under “Immunostaining of μ-opioid receptors following nerve injury”). Images were taken using light (μ-receptors in DRG) or fluorescent microscope with appropriate filters (μ-receptors in nerves and paws) and 20× objectives (Zeiss Axioskop 2), and the AxioVision program. Quantification was performed using the ImageJ graphic program (http://rsb.info.nih.gov/ij/).

Every second section of each serially cut DRG was stained for μ-receptors. The total number of all DRG neurons and of μ-receptor-immunoreactive neurons was counted, and the data expressed as percent of the total number of neurons per section. To assess the labeling intensity of μ-receptors in DRG neurons the images were converted to grayscale. For each image, the background intensity was assessed in three random areas not covered by neurons, averaged, and subtracted. Then, the μ-receptor-immunoreactive neurons were marked with a freehand selection tool. Their labeling intensity (in arbitrary units) was acquired and expressed as a mean staining intensity per section. For each group, six sections per animal were analyzed. The examiner was unaware of the experimental groups.

Every third section of each serially cut sciatic nerve was stained for μ-receptors. In injured nerves, the images were taken from three different areas in relation to ligatures: directly proximally (0–700 µm; proximal I), further proximally (700–1400 µm; proximal II), and directly distally (0–700 µm). Images of 700 µm-areas (corresponding to the injury site) in contralateral nerves of CCI mice, both sciatic nerves of sham-operated and naïve mice were also obtained. To measure the staining intensity, the upper and lower threshold density ranges were adjusted to encompass and match the immunoreactivity (red fluorescence) to provide an image with positive staining appearing in white pixels, and background staining in black pixels, for all images. A standardized box (0.2 mm^2^) was positioned over each 700 µm-area and the number of positively-stained (white) pixels per section was calculated. In parallel, the number of μ-receptor-stained fibers was quantified in each area, except for the proximal I region because strong immunoreactivity made the distinction of fibers difficult. For each animal, four sections from each nerve were analyzed. The examiner was unaware of the identity of nerve images from naïve and sham-operated mice, and of contralateral nerves from the CCI animals. It was not possible to fully blind injured nerves because the ligation site was visible. However, to minimize a possible bias, the images of the three areas (proximal I, proximal II, and distal) of injured nerves were blinded for quantification.

Every second section of each serially cut paw tissue was stained for μ-receptors. A rectangular box of constant size was placed over the immunostained area. The box size was based on the averaged area from four images showing μ-receptor-immunoreactive fibers, and calculated as 0.19 mm^2^. The total number of μ-receptor-immunoreactive fibers was counted per section. Additionally, to assess the labeling intensity of μ-receptors in sensory fibers, the images were converted to grayscale. For each image, the background intensity was assessed in three random areas not covered by fibers, averaged, and subtracted. Then, the μ-receptor-immunoreactive fibers were marked with a freehand selection tool, their labeling intensity (in arbitrary units) was acquired and expressed as a mean staining intensity per section. For each group, four sections per animal were analyzed by the examiner unaware of experimental groups. For all tissues, the data were first averaged for each animal and these values were used for statistical evaluations.

### Statistical analysis

The quantitative data are expressed as mean ± SEM. All data were normally distributed and of equal variance (as assessed by Kolmogorov-Smirnov test), and analyzed with one-way repeated measurements (RM) analysis of variance (ANOVA), followed by Bonferroni test. Differences were considered significant if p<0.05.

## Results

### μ-Opioid receptor antibody reveals specific staining in HEK 293 cells

**Figure 1:**
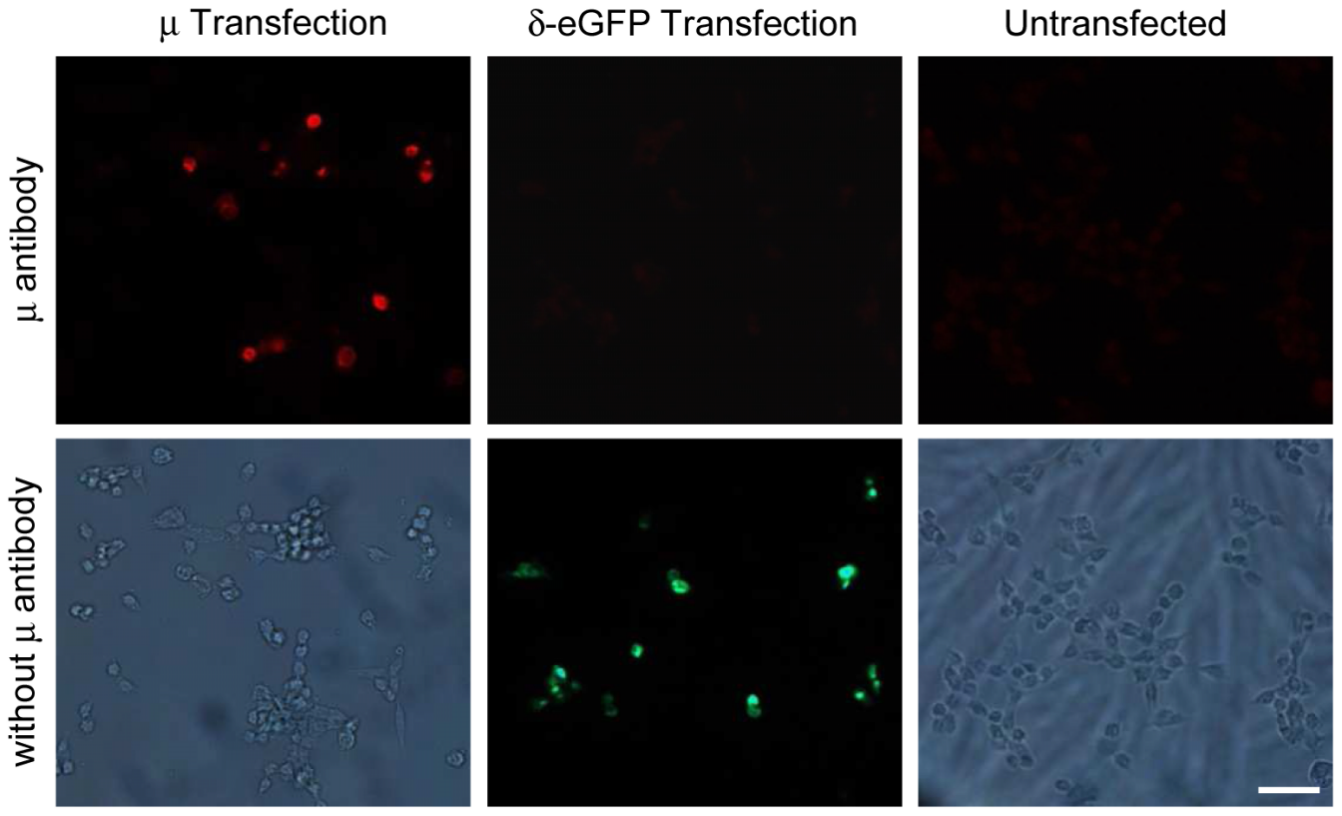
Specific staining of μ-opioid receptors in HEK 293 cells. Representative immunofluorescence images showing that the μ-receptor antibody only stains HEK 293 cells transfected with the mouse μ-opioid receptors, but not HEK 293 cells transfected with the mouse δ-opioid receptors coupled to eGFP, or in untransfected HEK 293 cells (upper panel: left, middle and right images, respectively). In the absence of μ-receptor antibody there was no staining in HEK 293 cells transfected with the μ-receptors or eGFP-coupled δ-receptors (showing only eGFP staining in green), or in untransfected HEK 293 cells (lower panel: left, middle and right images, respectively). Scale bar = 50 µm.

Staining with the μ-receptor antibody of HEK 293 cells transfected with the mouse μ-receptor revealed positively stained cells. In contrast, no positively stained cells were found in untransfected HEK 293 cells or HEK 293 cells transfected with the mouse δ-opioid receptor fused with eGFP, which appeared in green in the absence of the μ-receptor antibody (Fig. 1).

### μ-Opioid receptor antibody staining in DRG neurons

**Figure 2:**
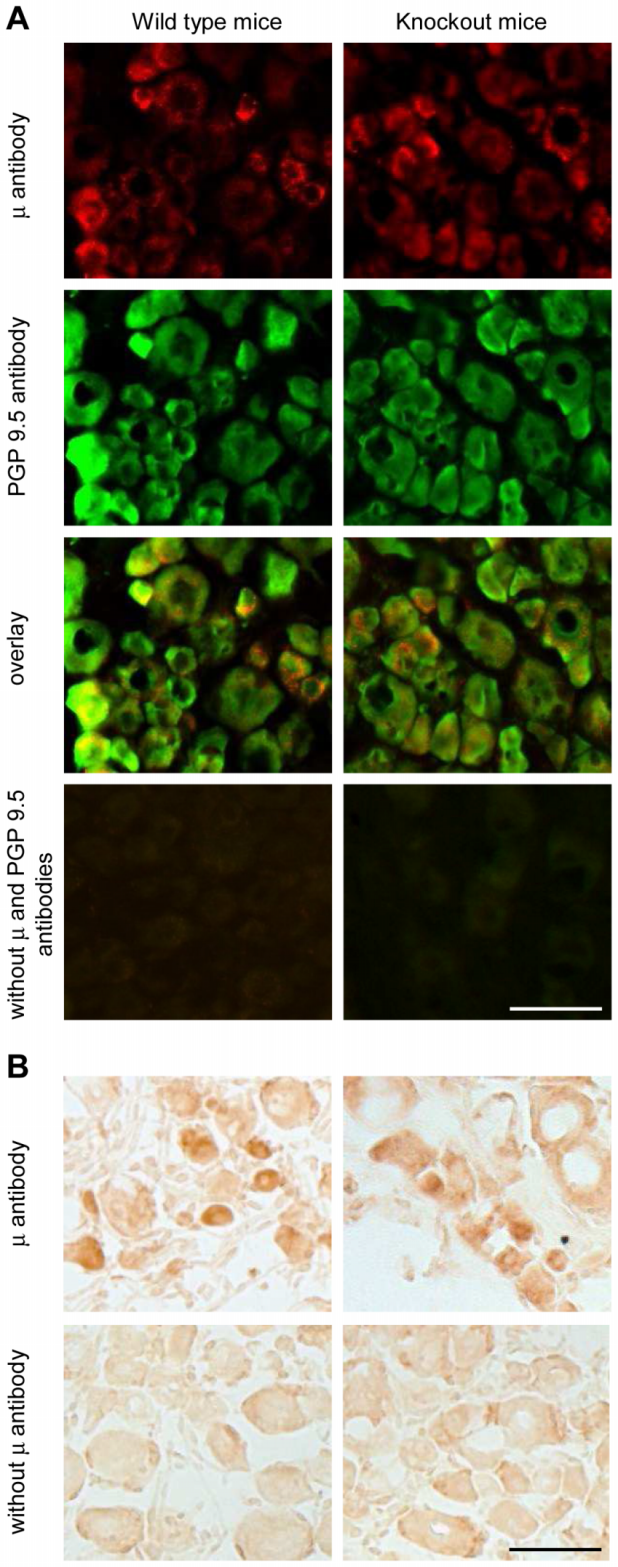
Non-specific staining of μ-opioid receptors in DRG neurons. (A) Representative double immunofluorescence images showing similar μ-receptor and PGP 9.5 staining in DRG neurons of wild type mice (left panel) and μ/δ/κ-opioid receptor knockout mice (right panel). Omission of antibodies to μ-receptors and PGP 9.5 resulted in no staining in both genotypes (bottom panel). (B) Representative DAB staining images showing similar μ-receptor labeling in DRG neurons of wild type mice (left image in the upper panel) and μ/δ/κ-opioid receptor knockout mice (right image in the upper panel). In the absence of μ-receptor antibody, some background DAB staining was visible in both genotypes (lower panel). Scale bars = 50 µm.

Specific staining of the μ-receptor antibody in HEK 293 cells prompted us to analyze the impact of nerve damage on μ-receptor expression in peripheral sensory pathways. On days 2 (Fig. S1) and 14 (data not shown) following nerve injury, we found numerous μ-receptor antibody-stained small- and medium-size DRG cells co-expressing CGRP which labels peptidergic C and A neurons, whereas few DRG cells co-expressed IB4 which binds non-peptidergic C neurons, or NF200 which marks myelinated A neurons, ipsilaterally to the nerve injury. Of all DRG neurons, 36±2% were positively stained with μ-receptor antibody in naïve animals, in agreement with previous studies [11], [15], [27]. Neither sham surgery nor CCI significantly changed the percentage of μ-receptor antibody-stained cells (p>0.05; Fig. S2A and B). The intensity of μ-receptor antibody labeling in DRG neurons was also not altered by the surgeries on days 2 and 14 (p>0.05; Fig. S2A and C). Preabsorption of the μ-receptor antibody with the μ-receptor immunizing peptide showed a lack of μ-receptor specific staining in DRG (Fig. S3); some background staining following preabsorption in the DAB staining image is similar to that seen in DAB experiments with the omission of the μ-receptor antibody (see Fig. 2B).

After completion of these experiments, we had access to opioid receptor knockout mice and decided to verify the μ-receptor antibody staining specificity. To ensure the targeting of neurons we also stained for the pan-neuronal marker PGP 9.5 with double immunofluorescence. Surprisingly, we found no difference in the μ-receptor antibody staining of DRG cells (co-labeled with PGP 9.5) between wild type and μ/δ/κ-opioid receptor knockout mice (Fig. 2A). Similarly, there was no difference between the two genotypes in the μ-receptor antibody labeling using DAB staining (Fig. 2B). Omission of antibodies to μ-receptors and PGP 9.5 showed no immunofluorescent staining (Fig. 2A), while some background staining was seen in the absence of μ-receptor antibody in experiments using DAB (Fig. 2B), both in wild type and μ/δ/κ-receptor knockout mice. Together, despite the positive outcome of the control experiments in HEK 293 cells (Fig. 1) and of the preabsorption experiments in DRG (Fig. S3), the staining in opioid receptor knockout mice clearly shows that the antibody did not specifically label μ-opioid receptors in the DRG, in our experimental conditions. Consequently, the analysis of μ-receptor expression in DRG neurons appears invalid (Fig. S1 and S2).

### μ-Opioid receptor antibody specifically labels peripheral neuronal processes in the sciatic nerve and the paw skin

**Figure 3:**
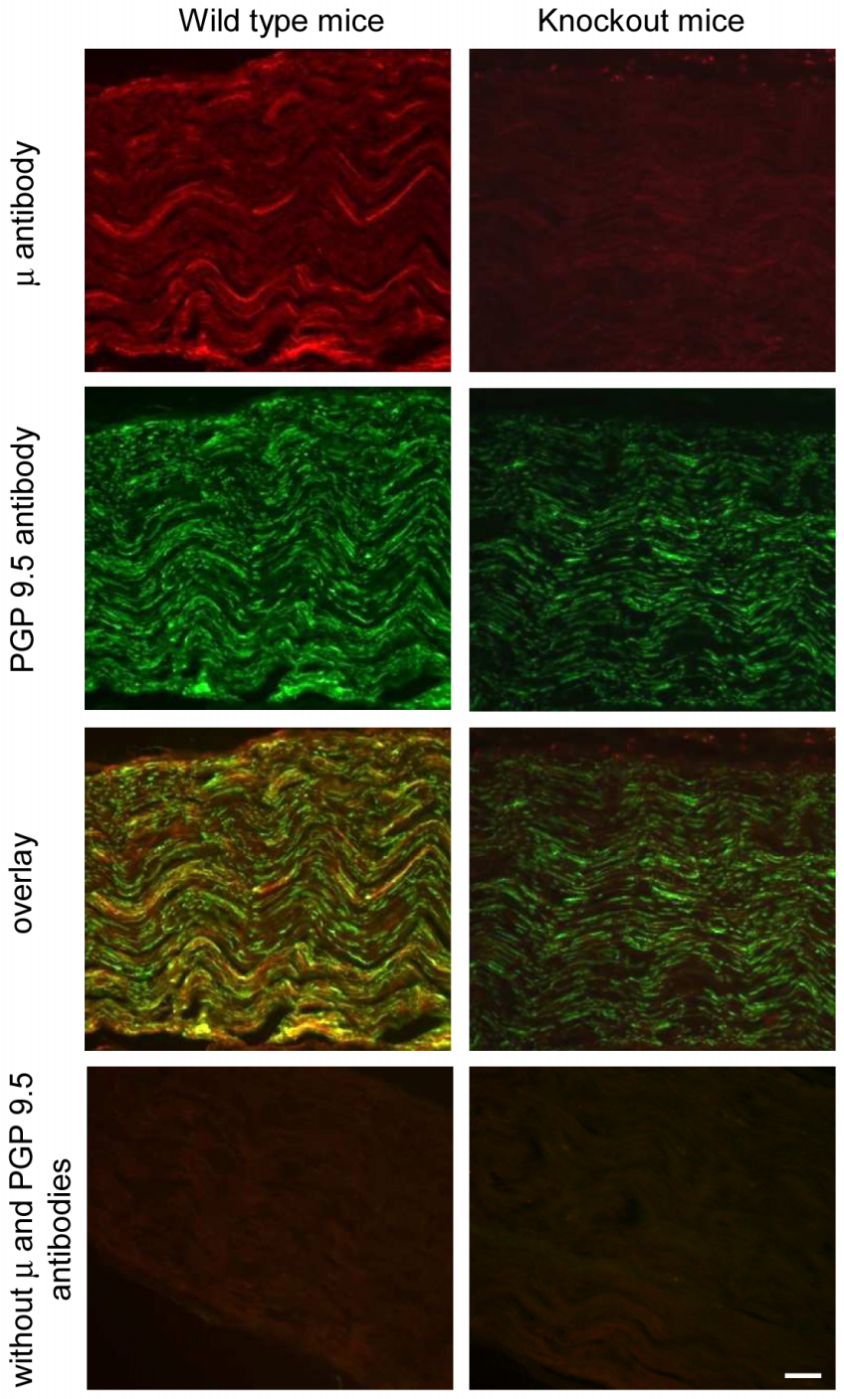
Specific staining of μ-opioid receptors in the sciatic nerve. Representative double immunofluorescence images showing μ-receptor and PGP 9.5 staining in the sciatic nerve of wild type mice (left panel), but only PGP 9.5 and no μ-receptor labeling in the μ/δ/κ-opioid receptor knockout mice (right panel). Omission of antibodies to μ-receptors and PGP 9.5 resulted in no staining in both genotypes (bottom panel). Scale bar = 50 µm.

**Figure 4:**
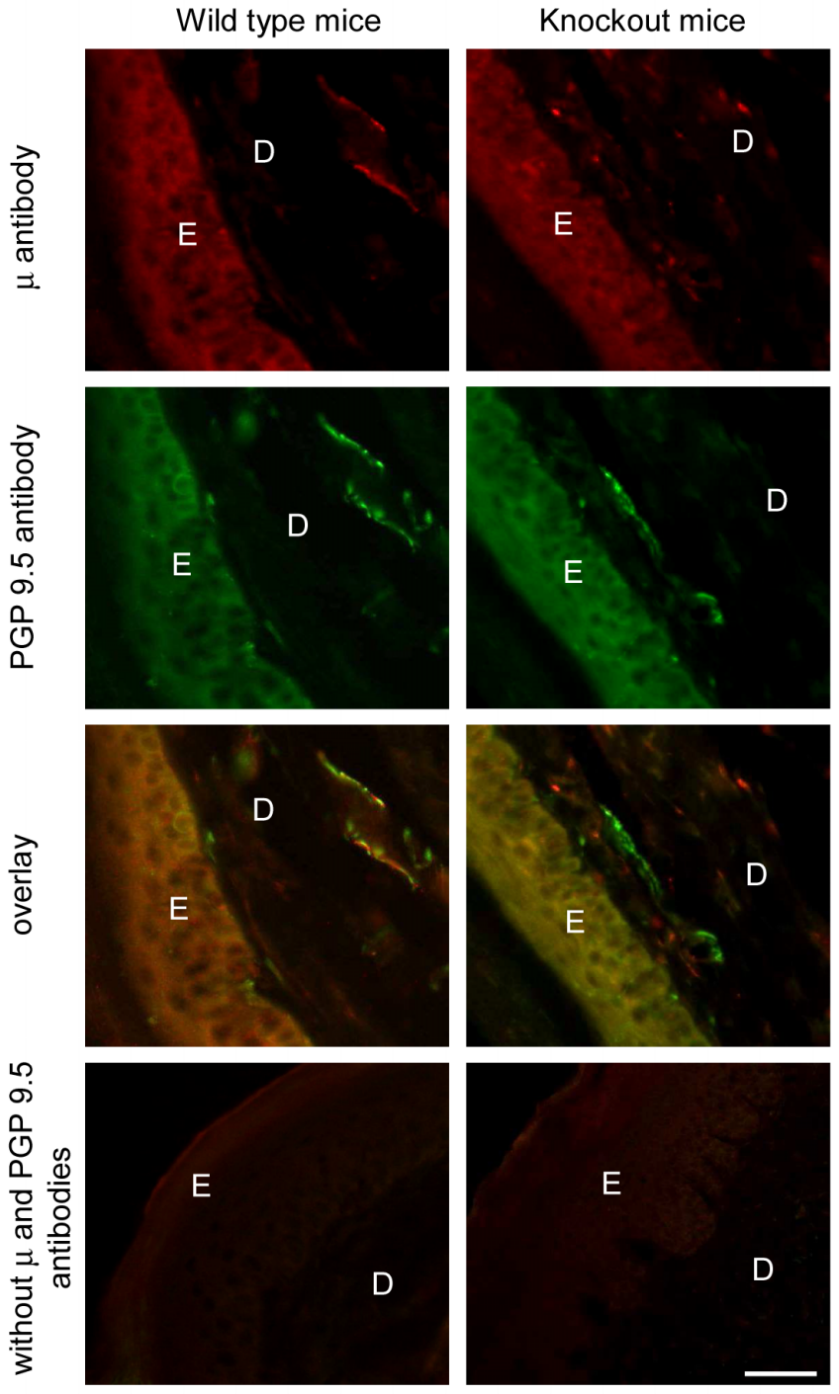
Specific staining of μ-opioid receptors in the hind paw skin dermis. Representative double immunofluorescence images showing μ-receptor and PGP 9.5 staining in the hind paw skin dermis of wild type mice (left panel), but only PGP 9.5 and no μ-receptor labeling in the μ/δ/κ-opioid receptor knockout mice (right panel). In contrast, the epidermis appears similarly stained by both antibodies in both genotypes. Omission of antibodies to μ-receptors and PGP 9.5 resulted in no staining in both genotypes (bottom panel). Scale bar = 50 µm. E, epidermis; D, dermis.

Interestingly, the μ-receptor antibody staining of neuronal processes in the sciatic nerve (Fig. 3) and in the hind paw skin dermis (Fig. 4) was present in wild type mice, but it was absent in μ/δ/κ-opioid receptor knockout mice. Thus, μ-receptor labeling overlaid the PGP 9.5 labeling in wild type mouse sciatic nerves and the paw skin dermis, whereas in μ/δ/κ-receptor knockout mice, the μ-receptor antibody staining was absent, but that of PGP 9.5 remained (Fig. 3 and 4). The skin epidermis was similarly labeled by both antibodies in both genotypes (Fig. 4). Omission of antibodies to μ-receptors and PGP 9.5 showed no staining in the nerve and paw skin of both genotypes (Fig. 3 and 4).

Because of this unexpected tissue-dependent staining specificity of μ-receptor antibody, we additionally tested spinal cord, which is known to be rich in μ-receptors. We found that μ-receptor and PGP 9.5 antibodies co-labeled the dorsal horn of the spinal cord in wild type mice, while in μ/δ/κ-receptor knockout mice only PGP 9.5 antibody staining was preserved (Fig. S4). Thus, it appears that the μ-receptor antibody specifically stains spinal cord, similarly to the sciatic nerve and paw skin dermis.

### Nerve injury enhances μ-opioid receptor immunoreactivity at the injured nerve trunk

**Figure 5:**
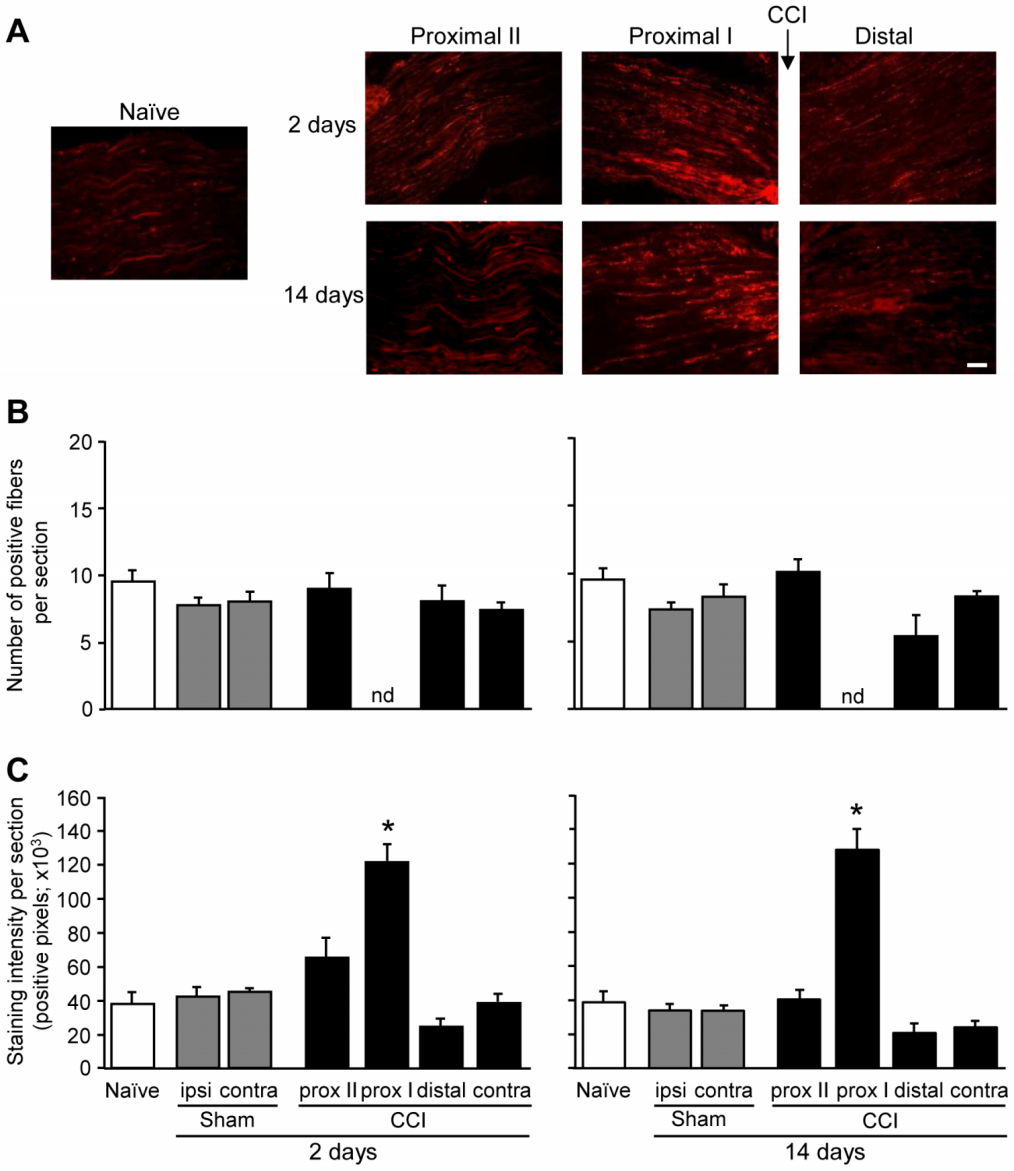
Elevation of μ-opioid receptor immunoreactivity at the injured nerve trunk. (A) Representative immunofluorescence images showing enhanced μ-receptor immunoreactivity directly proximally (0–700 µm; proximal I) to the CCI compared to regions more distant proximally (700–1400 µm; proximal II) and directly distally (0–700 µm) to the CCI in injured nerves. Corresponding region (700 µm-long) from the naïve nerve is also shown. Scale bar = 50 µm. (B) Quantitative analysis showing no significant alterations in the number of μ-receptor-immunoreactive neuronal fibers following surgeries (p>0.05; one-way RM ANOVA). (C) Quantitative analysis showing significantly increased intensity of μ-receptor staining (expressed as the number of positively-stained pixels) directly proximally (prox I) to the CCI (*p<0.05, versus all other conditions; one-way RM ANOVA, Bonferroni test). Experiments were performed in naïve mice and in mice on days 2 and 14 following CCI or sham surgery. Ipsi, ipsilateral; contra, contralateral; nd, not determined. Data are means ± SEM. N = 5–6 mice per group.

Specific μ-receptor labeling in peripheral neuronal processes prompted us to examine μ-receptor expression following nerve damage. In the sciatic nerve, representative immunofluorescence images show strong μ-receptor immunorectivity directly proximally to the nerve injury (Fig. 5A). Quantitative analysis revealed that there were no significant differences in the number of μ-receptor-immunoreactive sciatic nerve fibers among naïve, sham-operated, and CCI animals, on days 2 and 14 following surgeries (p>0.05; Fig. 5B). In contrast, we found a robust and significantly higher intensity of μ-receptor staining directly proximally to the ligatures on days 2 and 14 after CCI as compared to all other conditions (i.e. to areas located further proximally and distally to CCI, or to nerves of naïve and sham-operated mice) (p<0.05; Fig. 5C). There were no significant differences in the μ-receptor staining intensity between the two time points after surgeries (p>0.05; Fig. 5C). Preabsorption control experiments showed a lack of μ-receptor specific staining in the sciatic nerve and paw skin (Fig. S3).

### Nerve injury does not alter the μ-opioid receptor immunoreactivity in paws innervated by injured nerves

**Figure 6:**
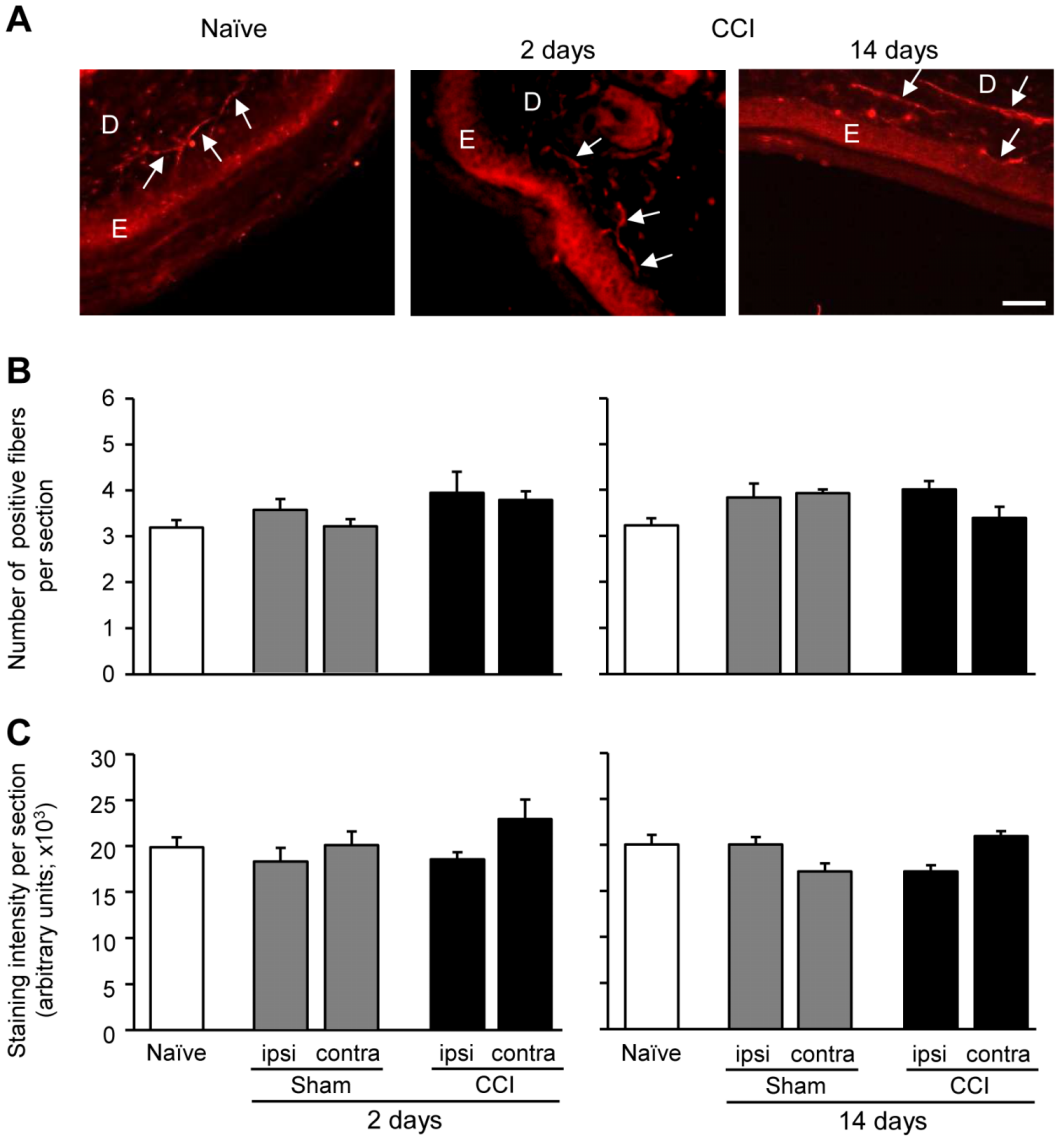
Unaltered μ-opioid receptor immunoreactivity in hind paws following nerve injury. (A) Representative immunofluorescence images showing μ-receptor-immunoreactive neuronal fibers (marked with arrows) in paws of naïve animals and paws innervated by injured nerves. Scale bar = 50 µm. E, epidermis; D, dermis. (B) Quantitative analysis showing no significant alterations in the number of μ-receptor-immunoreactive fibers following surgeries (p>0.05; one-way RM ANOVA). (C) Quantitative analysis showing no alterations in the intensity of μ-receptor staining (expressed in arbitrary units per section following surgeries (p>0.05, one-way RM ANOVA, Bonferroni test). Experiments were performed in naïve mice and in mice on days 2 and 14 following CCI or sham surgery. Ipsi, ipsilateral; contra, contralateral. Data are means ± SEM. N = 5–6 mice per group.

In the hind paw skin from naïve and CCI animals, μ-receptor-immunoreactive nerve fibers were found predominately within the dermis (Fig. 6A), in agreement with the labeling in Fig. 4. The number of μ-receptor-immunoreactive fibers was not significantly changed on days 2 and 14 after CCI or sham surgery (p>0.05; Fig. 6B). Likewise, there were no significant alterations in the labeling intensity of μ-receptors in sensory fibers after surgeries (p>0.05; Fig. 6C).

## Discussion

In this study, we focused on the impact of nerve injury on the μ-opioid receptor protein expression along the relevant peripheral neuronal pathways. We found that neuronal μ-receptor immunoreactivity was strongly enhanced at the nerve lesion site, while it was unaltered in the hind paw skin, at early (2 days) and later (14 days) neuropathy stages. In previous studies, μ-receptor agonists applied to paws innervated by injured nerves often moderately attenuated neuropathy-induced hypersensitivity [4]–[8] or were ineffective [9]–[12]. Notably, we have previously shown that activation of opioid receptors at the nerve damage site by opioid peptides derived from local immune cells inhibited CCI-induced mechanical hypersensitivity on days 2 and 14 [13], [14]. Similar time-course analgesia was also observed after exogenous μ-receptor agonist application at this site [15]. These findings suggest that opioid receptors at the site of axonal damage might be a promising target for neuropathic pain treatment.

The current debate about the lack of specificity of antibodies to opioid receptors and G protein-coupled receptors in general [22], [23], [28]–[30] prompted us to perform a detailed analysis of the μ-receptor antibody staining specificity. The antibody we used (Abcam; Ab10275) specifically stained μ-receptors in HEK 293 cells since only cells transfected with μ-receptors, but not with δ-receptors or untransfected cells, were positively labeled. Additionally, the lack of μ-receptor specific staining in the DRG, sciatic nerve, and paw skin in preabsorption control experiments suggests that the antibody selectively binds to its commercial immunizing peptide. However, the antibody did not specifically recognize native μ-receptors in the DRG, as it equally labeled DRG cells from wild type and μ/δ/κ-receptor knockout mice. This was true regardless whether we used immunofluorescence or DAB staining, in several independent experiments. Hence, although in our experiments the μ-receptor antibody stained a comparable percentage of DRG neurons (36%) and the same neuronal subpopulations (mostly CGRP- and some IB4- or NF200-positve cells), as in previous immunohistochemical studies assessing μ-receptor expression [11], [15], [27], [31], the labeled DRG proteins were apparently not μ-receptors or any other opioid receptors in our study.

Interestingly, however, the μ-receptor antibody specifically labeled μ-receptors in the neuronal processes of the sciatic nerve and paw skin dermis, and the spinal cord dorsal horn. This is supported by a co-staining of μ-receptors and the pan-neuronal marker PGP 9.5 in wild type mice, but only single PGP 9.5 staining in the nerve, skin dermis, and spinal cord of μ/δ/κ-receptor knockout animals. It seems unlikely that the antibody recognized δ- or κ-opioid receptors in sciatic nerve, paw skin, and spinal cord, because it is directed against an amino acid sequence that is absent in δ- or κ-receptors (see methods). Additionally, the lack of staining in δ-receptor transfected HEK cells and the unspecific staining in the DRG of triple μ/δ/κ-receptor knockout mice support the notion that the antibody does not cross-react with δ- or κ-receptors.

On the other hand, both μ-receptor and PGP 9.5 antibodies stained the skin epidermis in wild type and μ/δ/κ-receptor knockout animals. PGP 9.5 is abundantly present in the nervous system and is commonly used as an immunohistochemical marker for nerves [32], although melanocytes and Merkel cells also revealed PGP 9.5 immunoreactivity in human skin biopsies [33]. This might be a possible explanation for the immunoreactivity we found in the paw epidermis. Nevertheless, we cannot exclude non-specific PGP 9.5 labeling of the epidermis because this structure was non-specifically stained by the μ-receptor antibody in our study. Clearly, rigorous control experiments are needed when using commercial antibodies, despite the companies' claims on their specificity. Apparently, single bands of expected sizes on Western blots, provided on datasheets of commercial antibodies, or the disappearance of staining after preabsorption with immunizing peptides, are insufficient indicators for a specific labeling in immunohistochemistry [34], [35]. Our results support the use of animals genetically lacking the proteins of interest as the first choice criterion for specificity controls, in agreement with other studies [29], [30], [35], [36], since also data obtained from experiments using cell lines might not always be predictive for post-in vivo antibody staining. Moreover, our results indicate that antibodies might be even tissue- or tissue structure-selective. Interestingly, similar observations were made in another study, which examined antibodies to muscarinic receptors [34]. Thus, of 24 antibodies tested, only two antibodies were specific for muscarinic 2 receptors, as judged by the use of muscarinic 2 receptor knockout mice. Of these two antibodies, one was specific in 11 tissues but not in one (of 12 tissues examined), while the specificity of the other antibody depended on the batches [34]. Although there is no clear explanation for these differences, the results in our study and in that by Jositsch et al. [34] favor the examination of antibodies' specificity in each tissue of interest.

Thus, unfortunately, our detailed analysis aiming at the quantification of μ-receptor protein expression in the DRG following nerve damage appears inconclusive. Nevertheless, regardless of the opioid receptor expression in DRG cell bodies, the net protein level in peripheral sensory pathways might depend on injury-induced alterations in the receptor expression along the neuronal processes. Only one previous study analyzed μ-receptors directly at the nerve injury site, and reported an elevation of its immunoreactivity distally to the CCI [15]. Another study found decreased μ-receptor immunoreactivity proximally to the ligature in the sciatic nerve in animals with spinal nerve ligation (SNL), and suggested a reduced receptor anterograde transport in the sciatic nerve [17]. However, it is conceivable that μ-receptors accumulated at the SNL site (located proximally to the sciatic nerve ligation), but the receptor expression was not analyzed at the SNL site [17]. Moreover, since both studies used Western blot, the cellular sources of opioid receptors remain enigmatic [15], [17]. Using immunofluorescence and an antibody specifically staining μ-receptors in peripheral neuronal processes, we detected strongly enhanced μ-receptor immunoreactivity proximally to the CCI in the sciatic nerve. The colocalization of these μ-receptors with PGP 9.5 (this study) or with CGRP [13], suggests we labeled sensory fibers. The lack of changes in the number of fibers expressing μ-receptors indicates that the immunoreactivtiy increased only in fibers expressing μ-receptors already before nerve injury.

Explanation of the origin of the increased μ-receptor immunoreactivity at the nerve injury site is complicated by the lack of specific μ-receptor labeling in the DRG in our study and, therefore, can only be speculated. Thus, the increased μ-receptor immunoreactivity proximally to the nerve injury site could result from: (i) concomitantly increased (pre-existing or de novo) μ-receptor synthesis and anterograde transport leading to the receptor accumulation at the CCI site, if μ-receptor immunoreactivity in the DRG was enhanced, or (ii) a stronger rate of the receptor transport relative to its synthesis, if μ-receptor immunoreactivity in the DRG was unchanged or decreased. Alternatively, since mRNA of various proteins, including opioid receptors, have been found in axons [37], [38], the increased μ-receptor immunoreactivity at the nerve damage site could result from its locally enhanced synthesis. Additionally, a combination of several mechanisms cannot be excluded.

In paws innervated by injured nerves, former studies reported that μ-receptor immunoreactivity was elevated after CCI or partial nerve ligation, but decreased following SNL, using Western blot, without specifying cell types [17], [20], [21]. In contrast, we observed no changes in the number and the labeling intensity of sensory fibers expressing μ-receptors in the paw skin. Thus, methodological targeting of different cellular sources and/or nerve injury type might account for the variations among the studies.

## Conclusions

Because the antibody we used did not specifically stain μ-receptors in the DRG and it is unclear whether antibodies used to detect these receptors in other studies were rigorously assessed for the specificity [11], [15]–[17], [19], [20], it is currently difficult to judge whether the DRG μ-receptor protein levels are predictive for the peripheral μ-receptor-mediated analgesia in neuropathy. On the other hand, the specific labeling of μ-receptors in peripheral neuronal processes in our study suggests that the lack of increased neuronal μ-receptor immunoreactivity in the peripheral terminals might account for the moderate [4]–[8] or lacking [9]–[12] analgesic effects of μ-receptor agonists in paws. In contrast, since μ-receptor immunoreactivity was elevated at the site of axonal injury, targeting of these receptors might be more important for the control of neuropathic pain. Supporting this notion we have recently reported a stronger analgesic efficacy of opioids at the CCI site than in injured nerve-innervated paws [39].

Future studies should elucidate other mechanisms of peripheral opioid analgesia in neuropathy (e.g. ligand accessibility and affinity, receptor coupling and signaling). Notably, animal studies, which so far concentrated on μ-receptors on peripheral terminals of sensory neurons, have shown that these receptors can mediate a substantial portion of analgesia produced by systemically (intravenously, subcutaneously) injected μ-receptor-preferring agonists (morphine, loperamide) in neuropathic pain models [40], [41]. To strengthen the clinical application of these findings, a technology-oriented research is needed to find novel ways of drug delivery to the most relevant injured tissue [42]. For example, a recent study has shown that liposomes loaded with loperamide and conjugated with an antibody to intercellular adhesion molecule-1, injected intravenously, exclusively targeted damaged tissue and produced local analgesia in a model of inflammatory pain [43]. Clearly, opioid analgesics selectively acting in the most relevant injured peripheral tissue would be preferred for the lack of central and systemic adverse effects [2].

## Supporting Information

Figure S1 **Staining of μ-opioid receptor antibody and sensory neuron markers in the DRG.** Representative double immunofluorescence images showing that μ-receptor antibody predominantly stained DRG cells expressing CGRP (upper panel) and, to a lesser extend, cells expressing IB4 (middle panel) or NF200 (lower panel). Staining was performed in DRG ipsilateral to the injured nerve, at 2 days after CCI. Arrows indicate double-stained cells. Scale bar = 50 µm.
(TIF)

Figure S2 **Unaltered μ-opioid receptor antibody staining in the DRG following nerve injury.** (A) Representative DAB staining images showing μ-receptor antibody-stained neurons (marked with arrows) in DRG of naïve mice and in DRG ipsilateral to the nerve injury. Scale bar = 50 µm. (B) Quantitative analysis depicting no significant differences in the percentage of μ-receptor antibody-labeled DRG neurons following surgeries (p>0.05; one-way RM ANOVA). (C) Quantitative analysis showing no alterations in the intensity of μ-receptor antibody staining (expressed in arbitrary units per section in positively-stained DRG neurons) following surgeries (p>0.05, one-way RM ANOVA). Experiments were performed in naïve mice and in mice on days 2 and 14 following CCI or sham surgery. Ipsi, ipsilateral; contra, contralateral; nd, not determined. Data are means ± SEM. N = 5–6 mice per group.
(TIF)

Figure S3 **Preabsorption of μ-opioid receptor antibody with μ-receptor immunizing peptide in DRG, sciatic nerve, and hind paw skin.** (Upper panel) Representative DAB staining image (first from the left) and immunofluorescence images (second to fourth) showing μ-receptor staining in the DRG (first two images), the sciatic nerve (third image), and the paw skin (last image) in the presence of μ-receptor antibody. (Lower panel) Corresponding images showing the lack of μ-receptor staining following preabsorption of the μ-receptor antibody with μ-receptor immunizing peptide. Some background staining was visible in DAB staining image (see also Fig. 2B). Experiments were performed in tissues ipsilateral to nerve injury, at 2 days after CCI. Scale bars = 50 µm. E, epidermis; D, dermis.
(TIF)

Figure S4 **Specific staining of μ-opioid receptors in the spinal cord.** Representative double immunofluorescence images showing μ-receptor and PGP 9.5 staining in the spinal cord dorsal horn of wild type mice (left panel), but only PGP 9.5 and no μ-receptor labeling in the μ/δ/κ-opioid receptor knockout mice (right panel). Omission of antibodies to μ-receptors and PGP 9.5 resulted in no staining in both genotypes (bottom panel). Scale bar = 50 µm.
(TIF)
