# Supplementary figures and images for: μ-Opioid Receptor Antibody Reveals Tissue-Dependent Specific Staining and Increased Neuronal μ-Receptor Immunoreactivity at the Injured Nerve Trunk in Mice
Source: PLoS One. 2013 Nov 22;8(11):e79099. doi: 10.1371/journal.pone.0079099 (PMC3838372; doi:10.1371/journal.pone.0079099)

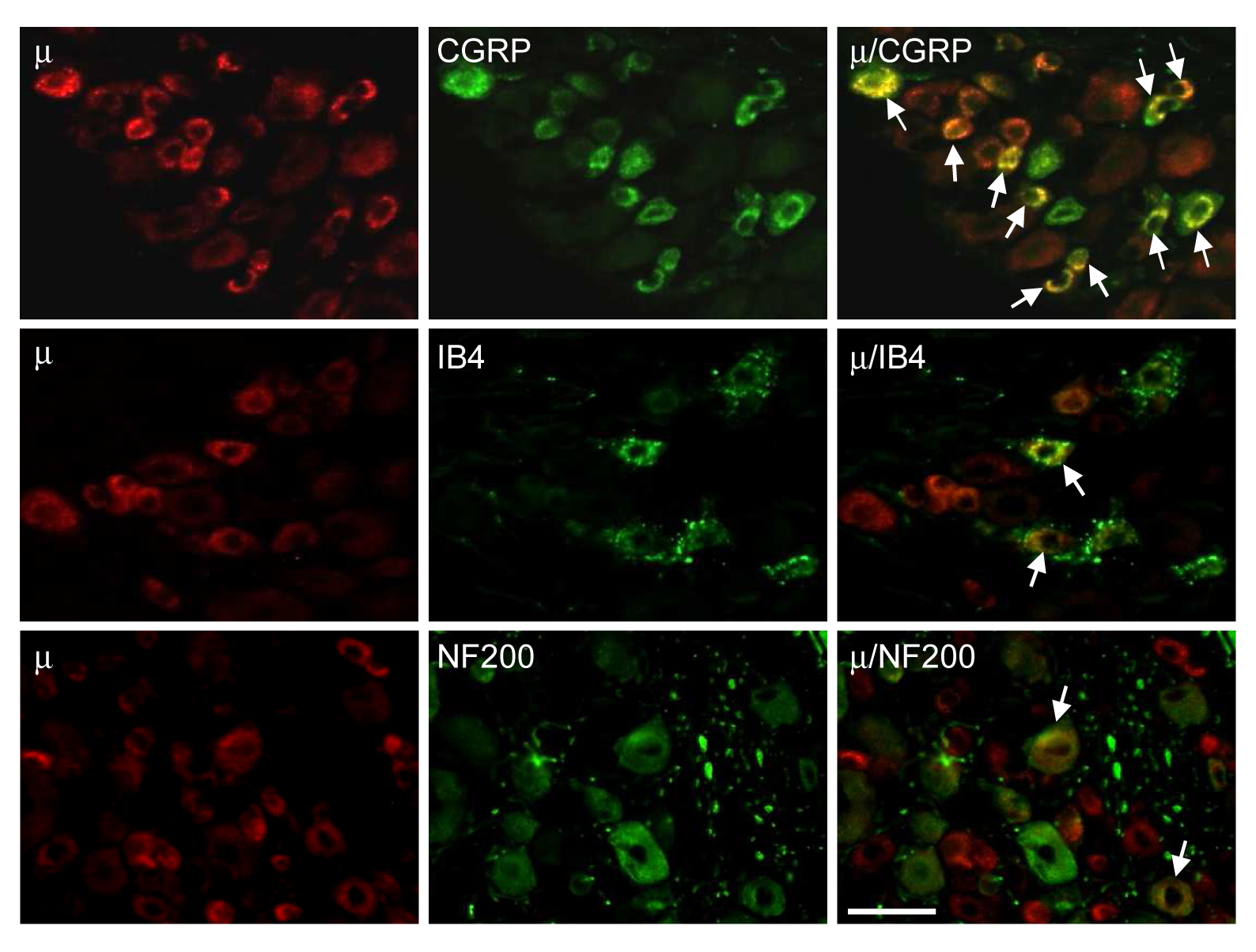

Supplement: Figure S1 — Staining of μ-opioid receptor antibody and sensory neuron markers in the DRG. Representative double immunofluorescence images showing that μ-receptor antibody predominantly stained DRG cells expressing CGRP (upper panel) and, to a lesser extend, cells expressing IB4 (middle panel) or NF200 (lower panel). Staining was performed in DRG ipsilateral to the injured nerve, at 2 days after CCI. Arrows indicate double-stained cells. Scale bar = 50 µm. (TIF) [file pone.0079099.s001.tif]

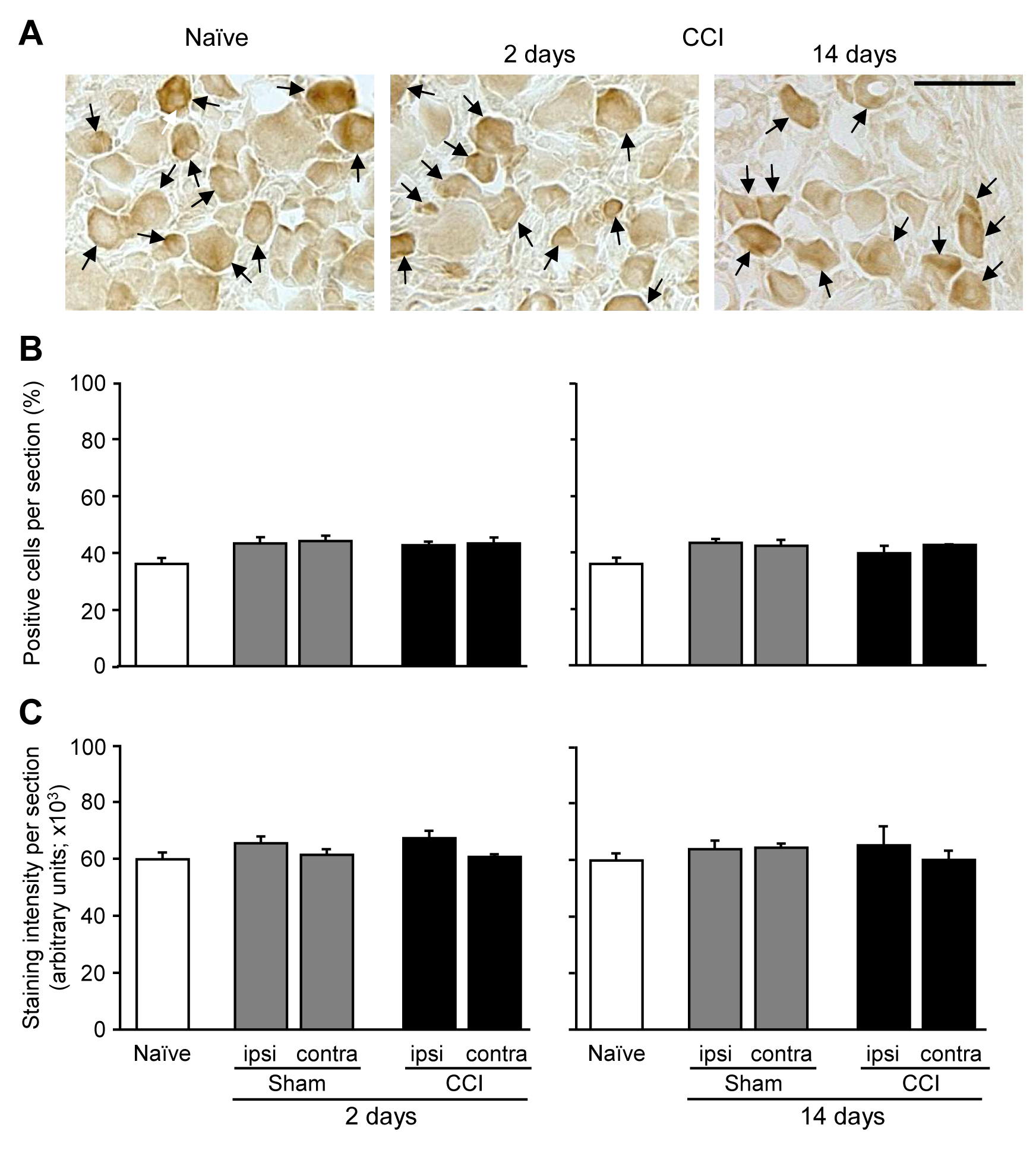

Supplement: Figure S2 — Unaltered μ-opioid receptor antibody staining in the DRG following nerve injury. (A) Representative DAB staining images showing μ-receptor antibody-stained neurons (marked with arrows) in DRG of naïve mice and in DRG ipsilateral to the nerve injury. Scale bar = 50 µm. (B) Quantitative analysis depicting no significant differences in the percentage of μ-receptor antibody-labeled DRG neurons following surgeries (p>0.05; one-way RM ANOVA). (C) Quantitative analysis showing no alterations in the intensity of μ-receptor antibody staining (expressed in arbitrary units per section in positively-stained DRG neurons) following surgeries (p>0.05, one-way RM ANOVA). Experiments were performed in naïve mice and in mice on days 2 and 14 following CCI or sham surgery. Ipsi, ipsilateral; contra, contralateral; nd, not determined. Data are means ± SEM. N = 5–6 mice per group. (TIF) [file pone.0079099.s002.tif]

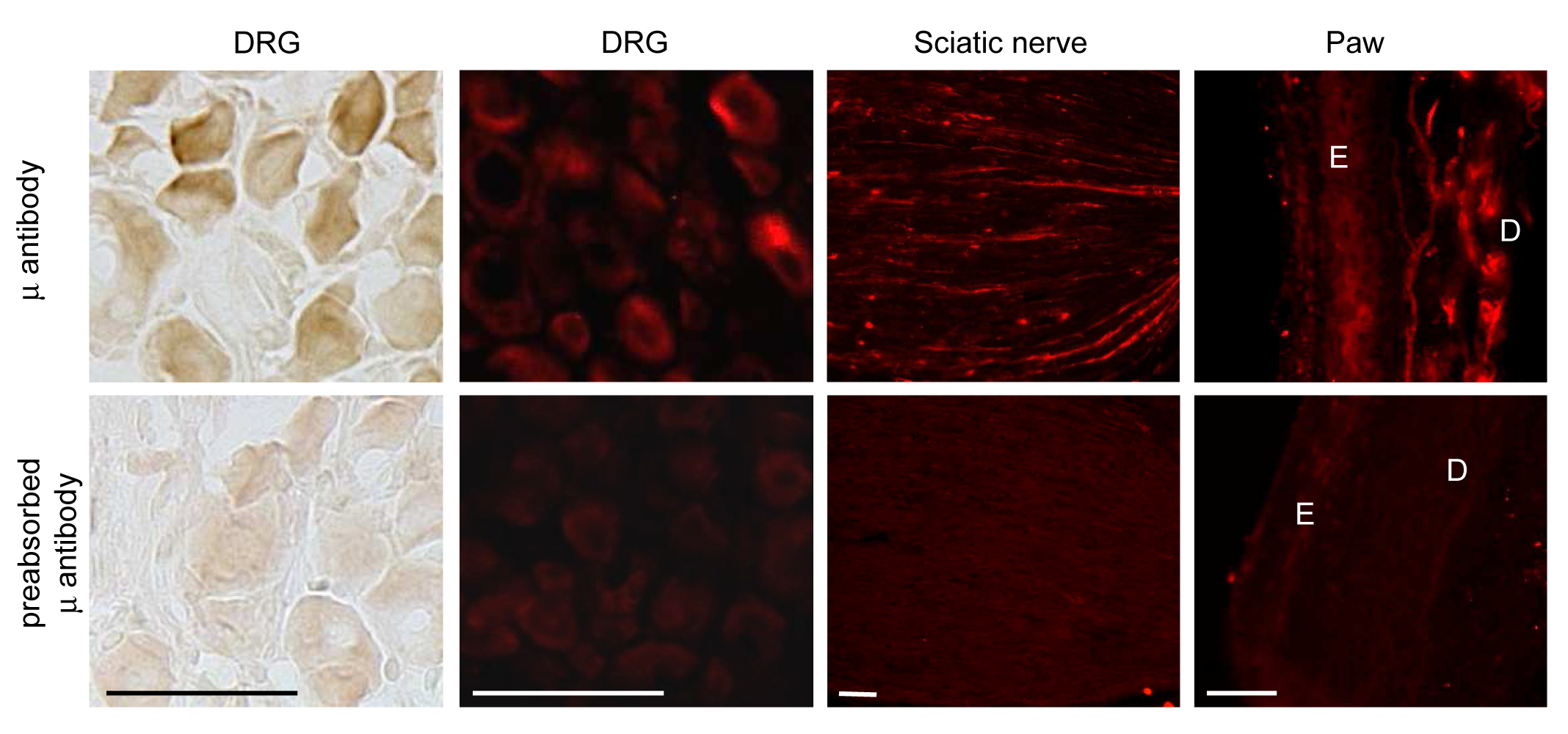

Supplement: Figure S3 — Preabsorption of μ-opioid receptor antibody with μ-receptor immunizing peptide in DRG, sciatic nerve, and hind paw skin. (Upper panel) Representative DAB staining image (first from the left) and immunofluorescence images (second to fourth) showing μ-receptor staining in the DRG (first two images), the sciatic nerve (third image), and the paw skin (last image) in the presence of μ-receptor antibody. (Lower panel) Corresponding images showing the lack of μ-receptor staining following preabsorption of the μ-receptor antibody with μ-receptor immunizing peptide. Some background staining was visible in DAB staining image (see also Fig. 2B). Experiments were performed in tissues ipsilateral to nerve injury, at 2 days after CCI. Scale bars = 50 µm. E, epidermis; D, dermis. (TIF) [file pone.0079099.s003.tif]

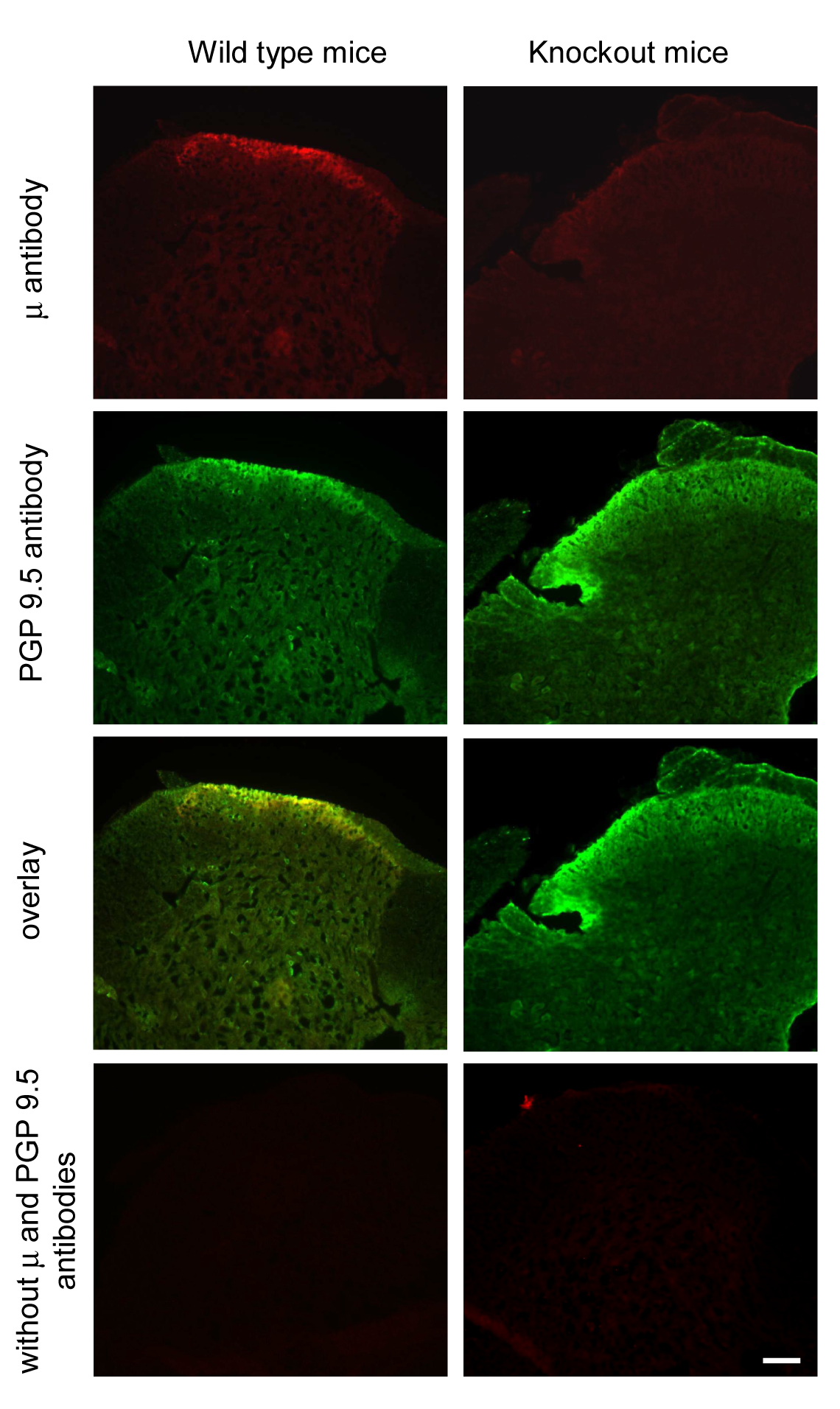

Supplement: Figure S4 — Specific staining of μ-opioid receptors in the spinal cord. Representative double immunofluorescence images showing μ-receptor and PGP 9.5 staining in the spinal cord dorsal horn of wild type mice (left panel), but only PGP 9.5 and no μ-receptor labeling in the μ/δ/κ-opioid receptor knockout mice (right panel). Omission of antibodies to μ-receptors and PGP 9.5 resulted in no staining in both genotypes (bottom panel). Scale bar = 50 µm. (TIF) [file pone.0079099.s004.tif]
